# Normal-Power-Logistic Distribution: Properties and Application in Generalized Linear Model

**DOI:** 10.1007/s41096-022-00143-4

**Published:** 2022-11-19

**Authors:** Matthew I. Ekum, Muminu O. Adamu, Eno E. E. Akarawak

**Affiliations:** 1 Department of Mathematical Sciences, College of Basic Sciences, Lagos State University of Science and Technology, Ikorodu, Lagos Nigeria; 2 Department of Statistics, Faculty of Science, University of Lagos, Akoka, Lagos Nigeria

**Keywords:** Bimodal, Exponential family, Generalized linear model, Normal-power$\lbrace$logistic$\rbrace$, Quantile function

## Abstract

The applications of Normal distribution in literature are verse, the new modified univariate normal power distribution is a new distribution which is adequate for modelling bimodal data. There are many data that would have been modelled by normal distribution, but because of their bimodality, they are not, since normal distribution is unimodal. In this paper, a new extension of the normal linear model called the normal-Power generalized linear model, derived from the T-Power$\lbrace$Logistic$\rbrace$ framework is presented. The statistical properties of the distribution and the proposed model were derived such as quantiles, median, mode, robust skewness, robust kurtosis and moment. The maximum likelihood estimation method was considered to obtain the unknown model parameters. Three real data sets were analyzed to demonstrate the flexibility and usefulness of the proposed model. The new model would be very useful as alternative in cases where skewed or bimodal response variables, which are not well fitted with normal linear model.

## Introduction

In probability and statistics, the power function and normal distributions are very useful in their individual applications. Not many authors have thought it to combine these two distributions. The normal distribution does not have a shape parameter, but power function has; while power function does not have a location parameter but normal has. Both are flexible, so combining them will produce a more flexible distribution. The power function distribution is the inverse of Pareto distribution (Dallas 1976). The power function distribution is a special model that can be formed or related to the uniform, Weibull, Kumaraswamy distributions. The power function distribution is considered one of the simplest and handy lifetime distributions.

Meniconi and Barry (1996) proposed the two-parameter power function distribution as a simple alternative to the exponential distribution when it comes to modelling failure data related to mortality rate and component failures. It is a special case of the beta distribution and one may sight the importance of the distribution in statistical tests such as the likelihood ratio test. The normal distribution on the other hand has been combined with other distribution to form a more flexible distribution, such as exponentiated-Normal (Gupta et al. 1998), Beta-Normal distribution (Eugene and Lee 2002), Gamma-Normal (GN) distribution (Zografos and Balakrishnan 2009), Kumaraswamy-Normal distribution (Cordeiro and de Castro 2011). Estimation of the power function parameters has been done by various authors, such as Zaka and Akhter (2013) .

Many classical distributions have been extensively used for modelling real data in many areas. However, in many situations; there is a clear need for extended forms of these distributions to improve the flexibility and goodness of fit of these distributions. For that reason, families of continuous distributions are developed by introducing one or more additional shape parameter(s) to the baseline distribution or by combining two or more distributions to produce new ones. Akarawak et al. (2013) described such new distributions as convoluted distributions. Some authors in recent years have developed frameworks used in combining these distributions to form new ones. A good example is the T-R$\lbrace$Y$\rbrace$ framework (Aljarrah et al. 2014). Since then, a lot of authors have been using it to develop flexible life time distributions that are hazard weighted functions of the baseline distributions. Weibull-Normal distribution (Alzaatreh et al. 2014) was one of the first normal distribution combined with other distribution using the T-R$\lbrace$Y$\rbrace$ framework. The Weibull power function distribution (Tahir et al. 2016) has a combination of power function and weibull distribution, using weibull distribution as a baseline distribution.

Among the authors that used the T-R$\lbrace$Y$\rbrace$ framework in 2016 includes (Alzaatreh et al. 2016) and Almagambetova et al. (2016). Okorie et al. (2017) proposed the modified power function distribution. Famoye et al. (2018) developed the Weibull-Normal$\lbrace$log-logistics$\rbrace$ distribution with the normal distribution as the baseline. Zubair et al. (2018) also used the framework to develop a new convoluted distribution. Other convoluted distributions developed using the framework include the reduced beta skewed laplace distribution (Arowolo et al. 2019); Odd Lomax-exponential$\lbrace$log-logistic$\rbrace$ distribution (Ogunsanya et al. 2019); exponentiated-exponential-Dagum$\lbrace$Lomax$\rbrace$ distribution (Ekum et al. 2020a); Lomax-Cauchy$\lbrace$uniform$\rbrace$ (Amalare et al. 2020); and Rayleigh-Cauchy$\lbrace$uniform$\rbrace$ (Ogunsanya et al. 2021).

The simplicity and usefulness of the power function distribution compelled the researchers to explore its further extensions, generalizations, and applications in different areas of science (Arshad et al. 2020; Ekum et al. 2020b). Recently, Gamma-Power$\lbrace$log-logistic$\rbrace$ distribution was proposed by Ekum et al. (2021) and demonstrated its usefulness in modelling skewed data. None of these study have combined normal and power function distribution, especially making power function distribution a baseline, except the normal-power$\lbrace$logistic$\rbrace$ distribution (NPLD) proposed in the work of Ekum et al. (2021). More so, many properties of the NPLD has not been defined and studied, and it has not been developed into a generalized linear model for predicting relationship in regression applications.

Predicting oil spillage is of a major interest to researchers in the field of Geo-science and geological statistics. In Nigeria, oil spillage is a major problem that have devastated the ecosystem and biodiversity of the Niger Delta region in Nigeria. The quantity of oil spilled may be estimated using the estimated spilled volume. The estimated spill volume of crude oil may be determined by the duration of clean-up (Whanda et al. 2016; Deinkuro et al. 2021). Also, researchers may want to know if they can predict their researchgate score using their citations and research items. These are emerging issues of interest to researchers, especially the ones in academics (Jordan (2015); O’Brien (2019)). More so, the COVID-19 mortality rate per population and the linear effect on the economic wellbeing of Nigerians is also worth to study. This is because, the GDP per capita can be affected by COVID-19 mortality. The COVID-19 factor is also an extra burden to the wellbeing of the people (Pak et al. (2020); Iluno et al. (2021)).

In literature, there are some modifications of the normal distribution, which produced multimodality (Kundu 2017), which has multiple modes with less number of parameters. The modification of the normal distribution developed by Kundu (2017) is a bivariate family of distributions, why the one developed here is a univariate family. More so, Kundu (2017) did not extend their distribution to generalized linear model. The motivation of this work is based on the modelling of independent variables in regression modelling that have bimodal features. Other authors such as Famoye et al. (2018), Kundu (2017), etc, had developed distributions that are bimodal but none has extended it to regression modelling. More so, real life problems like the crude oil spill volume, number of citations in research gate, GDP per Capita, etc are real variables which maximum values can be estimated, so they are bounded below by zero (non negative) and above by a real value, rather than infinity. Thus, a distribution with bounded support is necessary [0, $\lambda$], where $\lambda > 0$ is a real upper bound (Ekum et al. 2020b).

Thus, in this study, the aim is to adopt a novel univariate continuous probability distribution called the normal-power-logistics distribution NPLD, which was derived from the T-Power$\lbrace$logistic$\rbrace$ family proposed and studied by Ekum et al. (2021) and extends it into generalized linear model in order to solve real regression problems, where the dependent variables are bimodal and skewed with a known maximum value. The model has four parameters, two from the normal distribution and the other two from the power function distribution, which one of it is a shape parameter and the other is an upper bound parameter to control the extremes of the distribution. The scope covers different characterizations, properties, regression model, and parameter estimation of the NPLD model. The method of Maximum Likelihood Estimation (MLE) was used to estimate the model parameters. The importance of the new model was proved empirically using three real-life datasets. The proposed model would be very useful in engineering, medicine, and all fileds of life, where the dependent variable of interest to be predicted has bimodal features. It is expected to perform well when normal distribution fails to fit the data of interest.

## Materials and Methods

In this section, the theory and application of the proposed scheme are considered.

### The Method of Generating the T-R$\lbrace$Y$\rbrace$ Family of Distributions

The method of generating T-R$\lbrace$Y$\rbrace$ family of distributions is considered. The T-R$\lbrace$Y$\rbrace$ is a general approach for defining the *W*[*F*(*x*)] (a non-decreasing differentiable function) using the quantile function of a random variable *Y* in the T-X framework. Let *T*, *R* and *Y* be three random variables with cdf $F_{T}(x) = P(T \le x)$, $F_{R}(x) = P(R \le x)$ and $F_{Y}(x) = P(Y \le x)$ respectively, with corresponding pdf, $f_{T}(x)$, $f_{R}(x)$ and $f_{Y}(x)$. Also, $Q_{T}(x)$, $Q_{R}(x)$ and $Q_{Y}(x)$ are their corresponding quantile functions. It is assumed that *T* is supported on the interval (*a*, *b*) and *Y* is supported on the interval (*c*, *d*) such that $b > a$ and $d > c$ are real numbers.

### Important Operational Definition of Terms

The following definitions will be very useful in characterising the proposed model.

#### Definition 1

The cdf of T-$power\lbrace$logistic$\rbrace$ family of distributions proposed by Ekum et al. (2021) is given by

1 $$\begin{aligned} F_{X}(x) = F_{T}\left[ ln \left( \frac{x^{k}}{\lambda ^{k}-x^{k}} \right) \right] ; \, x < \lambda \end{aligned}$$

#### Definition 2

: The pdf of the T-$power\lbrace$logistic$\rbrace$ family is derived by taking the first derivative of $F_{X}(x)$ with respect to *x* and it is given by

2 $$\begin{aligned} f_{X}(x) = \frac{k\lambda ^{k}}{x\left( \lambda ^{k}-x^{k} \right) } f_{T}\left[ ln \left( \frac{x^{k}}{\lambda ^{k}-x^{k}} \right) \right] ; 0 \le x < \lambda \end{aligned}$$

Other definitions follow from Definitions (1) and (2)

#### Definition 3

: The survival function of the distribution from T-$power\lbrace$logistic$\rbrace$ family is given by

3 $$\begin{aligned} S_{X}(x) = 1 - F_{X}(x) \end{aligned}$$

#### Definition 4

: The hazard function of the distribution from T-$power\lbrace$logistic$\rbrace$ family is given by

4 $$\begin{aligned} h_{X}(x) = \frac{f_{X}(x)}{1-F_{X}(x)} \end{aligned}$$

#### Definition 5

: The cumulative hazard function of the distribution from T-$power\lbrace$logistic$\rbrace$ family is given by

5 $$\begin{aligned} H_{X}(x) = -log[1 - F_{X}(x)] \end{aligned}$$

#### Definition 6

: The reverse hazard function of the distribution from T-$power\lbrace$logistic$\rbrace$ family is given by

6 $$\begin{aligned} \tau _{X}(x) = \frac{f_{X}(x)}{F_{X}(x)} \end{aligned}$$

#### Definition 7

: The quantile function of T-$power\lbrace$logistic$\rbrace$ family is the inverse function of its cdf and it is given by

7 $$\begin{aligned} Q_{X}(p) = Q_{R}\lbrace F_{Y}[Q_{T}(p)]\rbrace \end{aligned}$$

where $Q_{T}(p) = F_{T}^{-1}(p)$. The quantile function is used in Monte Carlo method to simulate random variates of a distribution, and it is used to determine measures of partition. Several ways of quantile approximation when it is not in closed form are available in literature, of which quantile mechanics is one of such approach (Akagbue et al. 2017).

#### Definition 8

: The T-$power\lbrace$logistic$\rbrace$ family of distributions is derived from T-R$\lbrace$Y$\rbrace$ family proposed by Aljarrah et al. (2014) and Alzaatreh et al. (2014). The relationship among *T*, *R*, and *Y* are given thus: (i) $X = Q_{R}[F_{Y}(T)]$ in distribution, (ii) $Q_{X}(p) = Q_{R}\lbrace F_{Y}[Q_{T}(p)]\rbrace$, (iii) if $T=Y$ in distribution, then $X = R$ in distribution, and (iv) if $Y = R$ in distribution, then $X = T$ in distribution.

#### Definition 9

: Let *R* be a non-negative random variable with pdf $f_{R}(x)$, and let $E(R^{k})$ denote the $k^{th}$ moment of *R*, then

$$\begin{aligned} E(X^{k})\le E(R^{k}).E\lbrace [1- F_{Y}(T)]\rbrace \end{aligned}$$

where $E(X^{k})$ is the $k^{th}$ moment of the random variable, *X*; [$1- F_{Y}(.)$] is the survival function of the random variable *Y*, and *T* is the quantile values random variable *T* with respect to $f_{T}(x)$.

### Normal-Power function $\lbrace$logistic$\rbrace$ Model

The proposed model is a generalized linear model that takes the form

$$\begin{aligned} g(\mu _{i}) = \beta _{0} + \beta _{1}x_{1i} + \beta _{2}x_{2i} +...+ \beta _{p}x_{pi} \end{aligned}$$

where $g(\mu _{i})$ is the link function, and the right hand side is the linear predictor. Six goodness-of-fit criteria are used to compare the flexibility of the proposed model with other known models. The goodness-of-fit criteria are log-likelihood (LogL), Akaike Information Criterion (AIC), Kolmogorov-Smirnov statistic (D), Anderson-Darling statistic (A), Cramer-von Mises statistic ($\omega$) and Chi-square statistic ($\chi ^{2}$). See (Chen and Balakrishnan 1995) for detailed information on *A* and $\omega$. The lower the value of the criteria, the better the performance of the model. Also, to show the relationship between the observed dependent variable *y* and the predicted dependent variable ${\hat{y}}$, the coefficient of correlation is used. This shows the model that performs well if the correlation coefficient is high. It is assumed that the dependent variable *y* has a normal-power distribution.

#### Cumulative Distribution and Probability Density Functions of NPLD

Recall the cdf of T-$power\lbrace logistic \rbrace$ defined by Ekum et al. (2021) given in Definition (1) as

$$\begin{aligned} F_{X}(x) = F_{T}\left[ ln \left( \frac{x^{k}}{\lambda ^{k}-x^{k}} \right) \right] \end{aligned}$$

where $F_{T}[t]$ is the cdf random variable *T*. So, *T* can follow any known distribution.

If *T* follows a normal distribution with parameters $\mu$ and $\sigma$, then the pdf of *T* is given by

$$\begin{aligned} f_{T}(t) =\frac{1}{\sqrt{2 \pi \sigma ^{2}}} exp \left\{ -\frac{1}{2} \left( \frac{t - \mu }{\sigma }\right) ^{2}\right\} ; -\infty \le t \le \infty \end{aligned}$$

and the cdf of *T* is given by

$$\begin{aligned} F_{T}(t) = \frac{1}{2} \left[ 1+ erf \left( \frac{t-\mu }{\sigma \sqrt{2}}\right) \right] = \Phi \left( \frac{t-\mu }{\sigma } \right) \end{aligned}$$

Therefore

$$\begin{aligned} t = ln \left( \frac{x^{k}}{\lambda ^{k}-x^{k}} \right) \end{aligned}$$

So, put the value of *t* into $F_{T}(t)$ to have

$$\begin{aligned} F_{X}(x)= \Phi \left[ \frac{ln\left( \frac{x^{k}}{\lambda ^{k}-x^{k}} \right) -\mu }{\sigma } \right] \end{aligned}$$

So, put the value of *t* into $F_{T}(t)$ to have

8 $$\begin{aligned}&F_{X}(x)= \frac{1}{2}\left\{ 1 + erf\left[ \frac{ln\left( \frac{x^{k}}{\lambda ^{k}-x^{k}} \right) -\mu }{\sigma \sqrt{2}} \right] \right\} ; \nonumber \\&\quad \mu , \sigma , k, \lambda > 0; 0< x < \lambda \end{aligned}$$

where error function, *erf*(.) is given by

$$\begin{aligned} erf(x) = \frac{2}{\sqrt{\pi }}\int _{0}^{x}e^{-t^{2}}dt \end{aligned}$$

Equation (8) is the cdf of Normal-Power function $\lbrace$logistic$\rbrace$ distribution (NPLD)

The corresponding pdf of NPLD is given by taking the first derivative of $F_{X}(x)$ with respect to *x* and it is given by

9 $$\begin{aligned}&f_{X}(x)= \frac{k \lambda ^{k}}{x(\lambda ^{k}-x^{k})\sqrt{2\pi \sigma ^{2}}} exp\left\{ -\frac{1}{2\sigma ^{2}} \left[ ln\left( \frac{x^{k}}{\lambda ^{k}-x^{k}} \right) -\mu \right] ^{2} \right\} ; \nonumber \\&\quad \mu , \sigma , k, \lambda > 0; 0< x < \lambda \end{aligned}$$

where $\mu$ is a location parameter, *k* is a shape parameter, $\sigma$ is a scale parameter, and $\lambda$ doubles as a scale and upper bound parameter. A random variable *X* follows a NPLD if it can be defined as $X \sim NPLD(\mu , \sigma , k, \lambda )$.

**Fig. 1:**
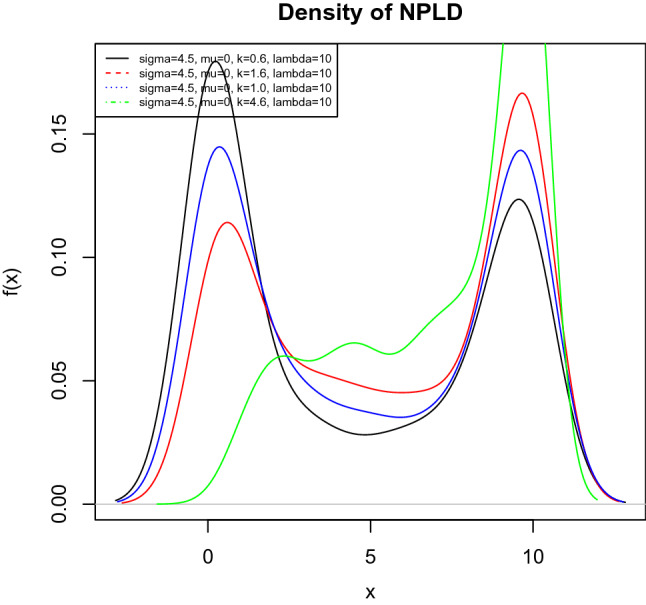
Probability Density Function with different parameters values showing bimodal features

Figure 1 is the pdf plot of NPLD, which shows that NPLD can be bimodal for some parameters values, skewed and kurtosis .

#### Useful Transformation

##### Lemma 2.1

If $X \sim NPLD(\mu , \sigma , k, \lambda )$, a random variable $W = ln \left( \frac{X^{k}}{\lambda ^{k}-X^{k}} \right)$ follows a normal distribution with parameters $\mu$ and $\sigma$, then the pdf of *W* is given by

$$\begin{aligned} f(w) =\frac{1}{\sqrt{2 \pi \sigma ^{2}}} exp \left\{ -\frac{1}{2} \left( \frac{w - \mu }{\sigma }\right) ^{2}\right\} \end{aligned}$$

##### Proof

Recall the pdf of NPLD in (9)

We want to show that random variable *W* follows a normal distribution with parameters $\mu$ and $\sigma$.

10 $$\begin{aligned} \int _{0}^{\lambda } f_{X}(x) dx = \int _{0}^{\lambda } \frac{k \lambda ^{k}}{x(\lambda ^{k}-x^{k})\sqrt{2\pi \sigma ^{2}}} \nonumber \\ \times exp\left\{ -\frac{1}{2\sigma ^{2}} \left[ ln\left( \frac{x^{k}}{\lambda ^{k}-x^{k}} \right) -\mu \right] ^{2} \right\} dx. \end{aligned}$$

By change of variable, let

11 $$\begin{aligned} w = ln \left( \frac{x^{k}}{\lambda ^{k}-x^{k}} \right) . \end{aligned}$$

Differentiating *w* with respect to *x*, and making *dx* the subject of the equation gives

12 $$\begin{aligned} dx = \frac{x(\lambda ^{k}-x^{k})}{k \lambda ^{k}}dw. \end{aligned}$$

Now, changing the support from *x* to that of *w*, we have

13 $$\begin{aligned} 0 \le x \le \lambda \quad \Rightarrow \quad -\infty \le w \le \infty \end{aligned}$$

It follows from inverse transformation and we have

14 $$\begin{aligned} f_{W}(w) = \frac{1}{\sqrt{2\pi \sigma ^{2}}} exp\left[ -\frac{1}{2} \left( \frac{w-\mu }{\sigma }\right) ^{2}\right] , \, -\infty \le w \le \infty \end{aligned}$$

is the pdf of normal distribution with parameters $\mu$ and $\sigma$. Equation (14) completes the proof. $\square$

From Lemma 2.1, it shows that the pdf of NPLD with parameters $(\alpha , \beta , k, \lambda )$ is a proper pdf. No further proof is needed.

#### Survival and Related Functions of NPLD

The survival function of NPLD is given by

15 $$\begin{aligned} S_{X}(x)= \frac{1}{2} \left\{ 1 - erf\left[ \frac{ln\left( \frac{x^{k}}{\lambda ^{k}-x^{k}} \right) -\mu }{\sigma \sqrt{2}} \right] \right\} , \end{aligned}$$

The hazard function of NPLD is given by

16 $$\begin{aligned} h_{X}(x)=\frac{2k\lambda ^{k}exp\left\{ -\frac{1}{2\sigma ^{2}} \left[ ln\left( \frac{x^{k}}{\lambda ^{k}-x^{k}} \right) -\mu \right] ^{2} \right\} }{x(\lambda ^{2}-x^{2})\sqrt{2\pi \sigma ^{2}}\left\{ 1 - erf\left[ \frac{ln\left( \frac{x^{k}}{\lambda ^{k}-x^{k}} \right) -\mu }{\sigma \sqrt{2}} \right] \right\} }, \end{aligned}$$

The cumulative hazard function of NPLD is given by

17 $$\begin{aligned} H_{X}(x)= -ln \left\{ \frac{1}{2} - \frac{1}{2}erf\left[ \frac{ln\left( \frac{x^{k}}{\lambda ^{k}-x^{k}} \right) -\mu }{\sigma \sqrt{2}} \right] \right\} \end{aligned}$$

The reverse hazard function of NPLD is given by

18 $$\begin{aligned} \tau _{X}(x)=\frac{2k\lambda ^{k}exp\left\{ -\frac{1}{2\sigma ^{2}} \left[ ln\left( \frac{x^{k}}{\lambda ^{k}-x^{k}} \right) -\mu \right] ^{2} \right\} }{x(\lambda ^{2}-x^{2})\sqrt{2\pi \sigma ^{2}}\left\{ 1 + erf\left[ \frac{ln\left( \frac{x^{k}}{\lambda ^{k}-x^{k}} \right) -\mu }{\sigma \sqrt{2}} \right] \right\} }. \end{aligned}$$

#### Quantile Function and Measures of Partition of NPLD

#### Quantile Function

##### Theorem 2.2

Let *X* be a random variable that follows NPLD with cdf $F_{X}(x)$, then the inverse function of the cdf, which is the quantile function exist, and it is given by

$$\begin{aligned} Q_{X}(p)= \lambda \left\{ \frac{e^{\left[ \mu + \sigma \Phi ^{-1}(p) \right] }}{1+e^{\left[ \mu + \sigma \Phi ^{-1}(p) \right] }}\right\} ^{1/k} \end{aligned}$$

##### Proof

Recall the cdf of NPLD given by

$$\begin{aligned} F_{X}(x)= \Phi \left[ \frac{ln\left( \frac{x^{k}}{\lambda ^{k}-x^{k}} \right) -\mu }{\sigma } \right] . \end{aligned}$$

Since $F_{X}(x)$ is a probability value, letting $p = F_{X}(x)$ gives

19 $$\begin{aligned} p= \Phi \left[ \frac{ln\left( \frac{x^{k}}{\lambda ^{k}-x^{k}} \right) -\mu }{\sigma } \right] . \end{aligned}$$

Solving for *x* gives

20 $$\begin{aligned} x = \left( \frac{\lambda ^{k}e^{\left[ \mu +\sigma \Phi ^{-1}(p)\right] } }{\left\{ 1 + e^{\left[ \mu +\sigma \Phi ^{-1}(p)\right] }\right\} }\right) ^{1/k} \end{aligned}$$

Equation (20) is the inverse function of the cdf of *X*, and it can be written as

21 $$\begin{aligned} Q_{X}(p)= \lambda \left\{ \frac{e^{\left[ \mu + \sigma \Phi ^{-1}(p) \right] }}{1+e^{\left[ \mu + \sigma \Phi ^{-1}(p) \right] }}\right\} ^{1/k} \end{aligned}$$

where $Q_{X}(p)$ is the quantile function of NPLD; $\Phi ^{-1}(p)$ is the inverse function of the cdf of standard normal distribution, and *p* is a probability value uniformly generated, that is, $P \sim U(0,1)$. Thus, Equation (21) completes the proof. $\square$

#### Measures of Partition

The quantile function can be used to derive all the measures of partition, such as, median, quartile, octile, decile and percentile.

The median of NPLD is

22 $$\begin{aligned} Q_{X}(p)= \lambda \left\{ \frac{e^{\left[ \mu + \sigma \Phi ^{-1}(0.5) \right] }}{1+e^{\left[ \mu + \sigma \Phi ^{-1}(0.5) \right] }}\right\} ^{1/k} \end{aligned}$$

But $\Phi ^{-1}(0.5) = 0$, so that

23 $$\begin{aligned} Q_{X}(p)= \lambda \left\{ \frac{e^{\mu }}{1+e^{\mu }}\right\} ^{1/k} \end{aligned}$$

The 1st quartile of NPLD, which is the same as the 25th percentile is given by

24 $$\begin{aligned} Q_{X}(0.25)= \lambda \left\{ \frac{e^{\left[ \mu + \sigma \Phi ^{-1}(0.25) \right] }}{1+e^{\left[ \mu + \sigma \Phi ^{-1}(0.25) \right] }}\right\} ^{1/k} \end{aligned}$$

But $\Phi ^{-1}(0.25) = -0.68$, so that

25 $$\begin{aligned} Q_{X}(0.25)= \lambda \left\{ \frac{e^{\left[ \mu - 0.68\sigma \right] }}{1+e^{\left[ \mu - 0.68\sigma \right] }}\right\} ^{1/k} \end{aligned}$$

The 3rd quartile of NPLD, which is the same as the 75th percentile is given by

26 $$\begin{aligned} Q_{X}(0.75)= \lambda \left\{ \frac{e^{\left[ \mu + \sigma \Phi ^{-1}(0.75) \right] }}{1+e^{\left[ \mu + \sigma \Phi ^{-1}(0.75) \right] }}\right\} ^{1/k} \end{aligned}$$

But $\Phi ^{-1}(0.75) = 0.68$, so that

27 $$\begin{aligned} Q_{X}(0.75)= \lambda \left\{ \frac{e^{\left[ \mu + 0.68\sigma \right] }}{1+e^{\left[ \mu + 0.68\sigma \right] }}\right\} ^{1/k} \end{aligned}$$

#### Skewness and Kurtosis of NPLD

#### Robust Measure of Skewness

By definition, the robust coefficient of skewness based on quantiles proposed by Bowley (1920) is given by

28 $$\begin{aligned} S_{k}= \frac{Q_{3}+Q_{1}-2Q_{2}}{Q_{3}-Q_{1}} \end{aligned}$$

where $Q_{i}$ is the *i*th quartile.

##### Theorem 2.3

Let *X* be a random variable that follows NPLD with quantile function $Q_{X}(p)$, then the skewness is robust, because it is a resistance measure, which is not affected by extreme value, $\lambda$.

##### Proof

Recall the median, 1st quartiles ($Q_{1}$) and 3rd quartile ($Q_{3}$) of NPLD given by

$$\begin{aligned} Q_{2}= \lambda \left\{ \frac{e^{\mu }}{1+e^{\mu }}\right\} ^{1/k}, \\ Q_{1}= \lambda \left\{ \frac{e^{\left[ \mu - 0.68\sigma \right] }}{1+e^{\left[ \mu - 0.68\sigma \right] }}\right\} ^{1/k} \end{aligned}$$

and

$$\begin{aligned} Q_{3}= \lambda \left\{ \frac{e^{\left[ \mu + 0.68\sigma \right] }}{1+e^{\left[ \mu + 0.68\sigma \right] }}\right\} ^{1/k} \end{aligned}$$

respectively.

Substituting the values of $Q_{2}$, $Q_{1}$ and $Q_{3}$ into (28) gives

29 $$\begin{aligned} S_{k}= \frac{\lambda \left\{ \frac{e^{\left[ \mu + 0.68\sigma \right] }}{1+e^{\left[ \mu + 0.68\sigma \right] }}\right\} ^{1/k}+\lambda \left\{ \frac{e^{\left[ \mu - 0.68\sigma \right] }}{1+e^{\left[ \mu - 0.68\sigma \right] }}\right\} ^{1/k}-2\lambda \left\{ \frac{e^{\mu }}{1+e^{\mu }}\right\} ^{1/k}}{\lambda \left\{ \frac{e^{\left[ \mu + 0.68\sigma \right] }}{1+e^{\left[ \mu + 0.68\sigma \right] }}\right\} ^{1/k}-\lambda \left\{ \frac{e^{\left[ \mu - 0.68\sigma \right] }}{1+e^{\left[ \mu - 0.68\sigma \right] }}\right\} ^{1/k}} \end{aligned}$$

Factorising out $\lambda$ gives

30 $$\begin{aligned} S_{k}= \frac{ \left\{ \frac{e^{\left[ \mu + 0.68\sigma \right] }}{1+e^{\left[ \mu + 0.68\sigma \right] }}\right\} ^{1/k}+ \left\{ \frac{e^{\left[ \mu - 0.68\sigma \right] }}{1+e^{\left[ \mu - 0.68\sigma \right] }}\right\} ^{1/k}-2 \left\{ \frac{e^{\mu }}{1+e^{\mu }}\right\} ^{1/k}}{ \left\{ \frac{e^{\left[ \mu + 0.68\sigma \right] }}{1+e^{\left[ \mu + 0.68\sigma \right] }}\right\} ^{1/k}- \left\{ \frac{e^{\left[ \mu - 0.68\sigma \right] }}{1+e^{\left[ \mu - 0.68\sigma \right] }}\right\} ^{1/k}} \end{aligned}$$

Equation (30) completes the proof, showing that it is free of $\lambda$. $\square$

#### Robust Measure of Kurtosis

Be definition, the robust coefficient of kurtosis based on quantiles proposed by Moors (1988) is given by

31 $$\begin{aligned} K_{u}= \frac{(E_{7}-E_{5})+(E_{3}-E_{1})}{E_{6}-E_{2}} \end{aligned}$$

where $E_{i}$ is the *i*th octile.

##### Theorem 2.4

Let *X* be a random variable that follows NPLD with quantile function $Q_{X}(p)$, then the kurtosis is robust, because it is a resistance measure, which is not affected by extreme value, $\lambda$.

##### Proof

Recall the quantile function of NPLD given by

$$\begin{aligned} Q_{X}(p)= \lambda \left\{ \frac{e^{\left[ \mu + \sigma \Phi ^{-1}(p) \right] }}{1+e^{\left[ \mu + \sigma \Phi ^{-1}(p) \right] }}\right\} ^{1/k} . \end{aligned}$$

The 1st octile ($E_{1}$) is derived thus

32 $$\begin{aligned}&E_{1}=Q_{X}(1/8)= \lambda \left\{ \frac{e^{\left[ \mu + \sigma \Phi ^{-1}(1/8) \right] }}{1+e^{\left[ \mu + \sigma \Phi ^{-1}(1/8) \right] }}\right\} ^{1/k} \nonumber \\&\quad = \lambda \left\{ \frac{e^{\left[ \mu - 1.15\sigma \right] }}{1+e^{\left[ \mu - 1.15\sigma \right] }}\right\} ^{1/k} \end{aligned}$$

The 2nd octile ($E_{2}$) is derived thus

33 $$\begin{aligned} E_{2}=Q_{X}(2/8)= \lambda \left\{ \frac{e^{\left[ \mu + \sigma \Phi ^{-1}(2/8) \right] }}{1+e^{\left[ \mu + \sigma \Phi ^{-1}(2/8) \right] }}\right\} ^{1/k} = Q_{1}. \end{aligned}$$

The 3rd octile ($E_{3}$) is derived thus

34 $$\begin{aligned}&E_{3}=Q_{X}(3/8)= \lambda \left\{ \frac{e^{\left[ \mu + \sigma \Phi ^{-1}(3/8) \right] }}{1+e^{\left[ \mu + \sigma \Phi ^{-1}(3/8) \right] }}\right\} ^{1/k} \nonumber \\&\quad = \lambda \left\{ \frac{e^{\left[ \mu - 0.32\sigma \right] }}{1+e^{\left[ \mu - 0.32\sigma \right] }}\right\} ^{1/k}. \end{aligned}$$

The 5th octile ($E_{5}$) is derived thus

35 $$\begin{aligned}&E_{5}=Q_{X}(5/8)= \lambda \left\{ \frac{e^{\left[ \mu + \sigma \Phi ^{-1}(5/8) \right] }}{1+e^{\left[ \mu + \sigma \Phi ^{-1}(5/8) \right] }}\right\} ^{1/k} \nonumber \\&\quad = \lambda \left\{ \frac{e^{\left[ \mu + 0.32\sigma \right] }}{1+e^{\left[ \mu + 0.32\sigma \right] }}\right\} ^{1/k}. \end{aligned}$$

The 6th octile ($E_{6}$) is derived thus

36 $$\begin{aligned} E_{6}=Q_{X}(6/8)= \lambda \left\{ \frac{e^{\left[ \mu + \sigma \Phi ^{-1}(6/8) \right] }}{1+e^{\left[ \mu + \sigma \Phi ^{-1}(5/8) \right] }}\right\} ^{1/k} = Q_{3}. \end{aligned}$$

The 7th octile ($E_{7}$) is derived thus

37 $$\begin{aligned}&E_{7}=Q_{X}(7/8)= \lambda \left\{ \frac{e^{\left[ \mu + \sigma \Phi ^{-1}(7/8) \right] }}{1+e^{\left[ \mu + \sigma \Phi ^{-1}(5/8) \right] }}\right\} ^{1/k} \nonumber \\&\quad = \lambda \left\{ \frac{e^{\left[ \mu + 1.15\sigma \right] }}{1+e^{\left[ \mu + 1.15\sigma \right] }}\right\} ^{1/k}. \end{aligned}$$

Substituting the values of $E_{1}$, $E_{2}$, $E_{3}$, $Q_{5}$, $E_{7}$ and $E_{7}$ into (31) gives

38 $$\begin{aligned} K_{u}= \frac{\lambda A -\lambda B+\lambda C -\lambda D}{\lambda \left\{ \frac{e^{\left[ \mu + 0.68\sigma \right] }}{1+e^{\left[ \mu + 0.68\sigma \right] }}\right\} ^{1/k}-\lambda \left\{ \frac{e^{\left[ \mu - 0.68\sigma \right] }}{1+e^{\left[ \mu - 0.68\sigma \right] }}\right\} ^{1/k}} . \end{aligned}$$

where $$A = \left\{ \frac{e^{\left[ \mu + 1.15\sigma \right] }}{1+e^{\left[ \mu + 1.15\sigma \right] }}\right\} ^{1/k}$$; $$B = \left\{ \frac{e^{\left[ \mu + 0.32\sigma \right] }}{1+e^{\left[ \mu + 0.32\sigma \right] }}\right\} ^{1/k}$$; $$C = \left\{ \frac{e^{\left[ \mu - 0.32\sigma \right] }}{1+e^{\left[ \mu - 0.32\sigma \right] }}\right\} ^{1/k}$$; $$D = \left\{ \frac{e^{\left[ \mu - 1.15\sigma \right] }}{1+e^{\left[ \mu - 1.15\sigma \right] }}\right\} ^{1/k}$$

Factorising out $\lambda$ gives

39 $$\begin{aligned} K_{u}= \frac{\ A - B + C - D }{ \left\{ \frac{e^{\left[ \mu + 0.68\sigma \right] }}{1+e^{\left[ \mu + 0.68\sigma \right] }}\right\} ^{1/k}-\left\{ \frac{e^{\left[ \mu - 0.68\sigma \right] }}{1+e^{\left[ \mu - 0.68\sigma \right] }}\right\} ^{1/k}}. \end{aligned}$$

Equation (39) completes the proof, showing that it is free of $\lambda$. $\square$

#### Mode of NPLD

##### Theorem 2.5

Let *X* be a random variable that follows NPLD with pdf $f_{X}(x)$, a differentiable function, then the mode is not unique and possibly bimodal for some parameter values.

##### Proof

Recall the pdf of NPLD given by

$$\begin{aligned} f_{X}(x)= \frac{k \lambda ^{k}}{x(\lambda ^{k}-x^{k})\sqrt{2\pi \sigma ^{2}}} exp\left\{ -\frac{1}{2\sigma ^{2}} \left[ ln\left( \frac{x^{k}}{\lambda ^{k}-x^{k}} \right) -\mu \right] ^{2} \right\} \end{aligned}$$

The mode can be derived by differentiating the pdf, equate to zero, and solve for *x*.

Using product rule

40 $$\begin{aligned} \frac{df_{X}(x)}{dx} =u\frac{dv}{dx}+v\frac{du}{dx} \end{aligned}$$

Let

41 $$\begin{aligned} u = \frac{k \lambda ^{k}}{x(\lambda ^{k}-x^{k})} \end{aligned}$$

and

42 $$\begin{aligned} v = \frac{1}{\sqrt{2\pi \sigma ^{2}}}exp\left\{ -\frac{1}{2\sigma ^{2}} \left[ ln\left( \frac{x^{k}}{\lambda ^{k}-x^{k}} \right) -\mu \right] ^{2} \right\} \end{aligned}$$

Differentiating *u* with respect to *x* gives

43 $$\begin{aligned} \frac{du}{dx} = \frac{(k+1)x^{k}-kx^{k}}{x(\lambda ^{k}-x^{k} )^{2}} \end{aligned}$$

Differentiating *v* with respect to *x* gives

44 $$\begin{aligned}&\frac{dv}{dx} = -\frac{k\lambda ^{k}}{x(\lambda ^{k}-x^{k} )\sqrt{2\pi \sigma ^{2}}}exp\left\{ -\frac{1}{2}\left[ \frac{ln\left( \frac{x^{k}}{\lambda ^{k}-x^{k}} \right) - \mu }{\sigma } \right] ^{2} \right\} \nonumber \\&\quad \times \left[ \frac{ln\left( \frac{x^{k}}{\lambda ^{k}-x^{k}} \right) - \mu }{\sigma ^{2}} \right] \end{aligned}$$

Inserting (41), (42), (43) and (44) into (40) and equating to zero gives

45 $$\begin{aligned} \sigma ^{2}(k+1)x^{k+1}-k\lambda ^{2k}\sigma ^{2}x-k^{2}\lambda ^{2k}ln\left( \frac{x^{k}}{\lambda ^{k}-x^{k}} \right) +\mu k^{2}\lambda ^{2k} = 0 \end{aligned}$$

The solution to (45) is the mode of NPLD.

Now, assume that $\sigma =k=\lambda =1$ and $\mu =0$, (45) becomes

46 $$\begin{aligned} 2x^{2}-x-ln\left( \frac{x}{1-x} \right) = 0; \, 0< x < 1 \end{aligned}$$

It is obvious from (46) that the mode of NPLD is not unique and it is possibly bimodal. The value of the shape parameter determines if it is bimodal or multi-modal. If *k* = 1, it is bimodal, if *k* = 2, it will have 3 peaks, if *k* = 3, it will have 4 peaks. However, some of these peaks might not be visible or obvious graphically because there can be repeated roots of the polynomial equation. The resulting equation for the mode is a polynomial of order *k*+1 as shown in equation (45). $\square$

#### Series Expansion of NPLD

##### Theorem 2.6

Let *X* be a random variable that follows NPLD with parameters $\mu , \sigma , k, \lambda$, the pdf of *X*, $f_{X}(x)$, is a weighted pdf of power function distribution with parameters *k* and $\lambda$, that is,

47 $$\begin{aligned} f_{X}(x)=\Psi f_{R}(x) \end{aligned}$$

where $f_{R}(x)$ is the pdf of power function distribution, and $\Psi$ is the weight.

##### Proof

Recall the pdf of NPLD given in (9). Given the following series expansions

48 $$\begin{aligned}&exp(y)= \sum _{i=0}^{\infty }\frac{y^{i}}{i!}, \end{aligned}$$

49 $$\begin{aligned}&(y+a)^{n}= \sum _{i=0}^{\infty } \begin{pmatrix} n \\ j \end{pmatrix} a^{n-j}y^{j}, \end{aligned}$$

50 $$\begin{aligned}&\left[ ln\left( \frac{x^{k}}{\lambda ^{k}-x^{k}} \right) -\mu \right] ^{2i} = \sum _{j=0}^{\infty } \begin{pmatrix} 2i \\ j \end{pmatrix} (-\mu )^{2i-j} \nonumber \\&\times \left[ ln\left( \frac{x^{k}}{\lambda ^{k}-x^{k}} \right) \right] ^{j}, \end{aligned}$$

51 $$\begin{aligned}&ln(y) = \sum _{l=0}^{\infty }\frac{(-1)^{l-1}(y-1)^{l}}{l} \end{aligned}$$

52 $$\begin{aligned}&ln\left( \frac{x^{k}}{\lambda ^{k}-x^{k}} \right) = \sum _{l=0}^{\infty }\frac{(-1)^{l-1}\left[ \left( \frac{x^{k}}{\lambda ^{k}-x^{k}} \right) -1\right] ^{l}}{l}, \end{aligned}$$

53 $$\begin{aligned}&(y+a)^{n}= \sum _{m=0}^{\infty } \begin{pmatrix} n \\ m \end{pmatrix} a^{n-m}y^{m}, \nonumber \\&\left[ \left( \frac{x^{k}}{\lambda ^{k}-x^{k}} \right) -1\right] ^{lj}= \sum _{i=0}^{\infty } \begin{pmatrix} lj \\ m \end{pmatrix} (-1)^{lj-m} \end{aligned}$$

54 $$\begin{aligned}&\times \left[ \left( \frac{x^{k}}{\lambda ^{k}-x^{k}} \right) \right] ^{m}, \end{aligned}$$

55 $$\begin{aligned}&(y+a)^{n}= \sum _{s=0}^{\infty } \begin{pmatrix} n \\ s \end{pmatrix} a^{n-s}y^{s}, \end{aligned}$$

56 $$\begin{aligned}&(\lambda ^{k}-x^{k})^{-(m+1)}= \sum _{s=0}^{\infty } \begin{pmatrix} -(m+1) \\ s \end{pmatrix} (\lambda ^{k})^{-(m+1)-s}(-x^{k})^{s}. \end{aligned}$$

Inserting (48–56) into the pdf of NPLD in (9) gives

57 $$\begin{aligned} f_{X}(x)=\frac{1}{\sqrt{2\pi \sigma ^{2}}} \sum _{i=j=l=m=s=0}^{\infty } (-1)^{3i-2j+2lj-m+s}\frac{1}{(m+s)} \nonumber \\ \times \begin{pmatrix} lj \\ m \end{pmatrix} \begin{pmatrix} 2i \\ j \end{pmatrix} \begin{pmatrix} -(m+1) \\ s \end{pmatrix} \frac{\mu ^{2i-j}k(m+s)x^{k(m+s)-1}}{2^{i}l^{j}\lambda ^{k(m+s)}\sigma ^{2i}i!}. \end{aligned}$$

Equation (57) is the series expansion form of NPLD pdf.

Now, let

$$\begin{aligned} \Psi =\frac{1}{\sqrt{2\pi \sigma ^{2}}} \sum _{i=j=l=m=s=0}^{\infty } (-1)^{3i-2j+2lj-m+s}\frac{1}{(m+s)} \begin{pmatrix} lj \\ m \end{pmatrix} \\ \times \begin{pmatrix} 2i \\ j \end{pmatrix} \begin{pmatrix} -(m+1) \\ s \end{pmatrix} \frac{\mu ^{2i-j}}{2^{i}l^{j}\sigma ^{2i}i!}. \end{aligned}$$

58 $$\begin{aligned}&\quad f_{X}(x)=\Psi \frac{k(m+s)x^{k(m+s)-1}}{\lambda ^{k(m+s)}}. \end{aligned}$$

If $$m=s=0$$, then

59 $$\begin{aligned}&\Psi =\frac{1}{\sqrt{2\pi \sigma ^{2}}} \sum _{i=j=l=0}^{\infty } (-1)^{3i-2j+2lj} \begin{pmatrix} 2i \\ j \end{pmatrix} \frac{\mu ^{2i-j}}{2^{i}l^{j}\sigma ^{2i}i!}. \nonumber \\&\quad f_{X}(x)=\Psi \frac{kx^{k-1}}{\lambda ^{k}}. \end{aligned}$$

where $f_{R}(x)$ is the pdf of power function distribution. Hence, Equation (59) completes the proof. $\square$

#### Moment of NPLD

Let *X* be a continuous random variable with pdf $f_{X}(x)$, the rth moment is given by

60 $$\begin{aligned} E(X^{r}) = \int f_{X}(x) dx \end{aligned}$$

Recall the series expansion form of NPLD pdf given as

$$\begin{aligned} f_{X}(x)=\frac{1}{\sqrt{2\pi \sigma ^{2}}} \sum _{i=j=l=m=s=0}^{\infty } (-1)^{3i-2j+2lj-m+s}\frac{1}{(m+s)} \\ \times \begin{pmatrix} lj \\ m \end{pmatrix} \begin{pmatrix} 2i \\ j \end{pmatrix} \begin{pmatrix} -(m+1) \\ s \end{pmatrix} \frac{\mu ^{2i-j}k(m+s)x^{k(m+s)-1}}{2^{i}l^{j}\lambda ^{k(m+s)}\sigma ^{2i}i!}. \end{aligned}$$

Inserting $f_{X}(x)$ into Equation (60) gives

61 $$\begin{aligned} E(X^{r}) = \int x^{r}f_{X}(x) dx \end{aligned}$$

Note that

62 $$\begin{aligned} \sum \int = \int \sum \end{aligned}$$

So that

63 $$\begin{aligned}&E(X^{r})= \sum _{i=j=l=m=s=0}^{\infty }\frac{(-1)^{3i-2j+2lj-m+s}}{2^{i}l^{j}i!} \begin{pmatrix} lj \\ m \end{pmatrix} \nonumber \\&\quad \times \begin{pmatrix} 2i \\ j \end{pmatrix} \begin{pmatrix} -(m+1) \\ s \end{pmatrix} \frac{1}{\sqrt{2\pi \sigma ^{2}}} \frac{\mu ^{2i-j}k}{\lambda ^{k(m+s)}\sigma ^{2i}}\left( \frac{\lambda ^{r+k}}{r+k}\right) . \end{aligned}$$

Let

64 $$\begin{aligned}&\psi _{i,j,l,m,s}= \sum _{i=j=l=m=s=0}^{\infty }\frac{(-1)^{3i-2j+2lj-m+s}}{2^{i}l^{j}i!} \begin{pmatrix} lj \\ m \end{pmatrix} \begin{pmatrix} 2i \\ j \end{pmatrix} \nonumber \\&\quad \times \begin{pmatrix} -(m+1) \\ s \end{pmatrix}. \end{aligned}$$

So that

65 $$\begin{aligned} E(X^{r})= \psi _{i,j,l,m,s} \frac{1}{\sqrt{2\pi \sigma ^{2}}} \frac{\mu ^{2i-j}k}{\sigma ^{2i}}\left[ \frac{\lambda ^{r+k-k(m+s)}}{r+k}\right] . \end{aligned}$$

Equation (65) is the rth moment of GPLD.

#### Mean of NPLD

If $r=1$, then (65) becomes

66 $$\begin{aligned} E(X)= \psi _{i,j,l,m,s} \frac{1}{\sqrt{2\pi \sigma ^{2}}} \frac{\mu ^{2i-j}k}{\sigma ^{2i}}\left[ \frac{\lambda ^{1+k-k(m+s)}}{k+1}\right] . \end{aligned}$$

Note that if $i=j=l=m=s=0$, then Equation (66) becomes

67 $$\begin{aligned} E(X)= \frac{k\lambda ^{k+1}}{(k+1)\sqrt{2\pi \sigma ^{2}}}. \end{aligned}$$

### Maximum Likelihood Estimation (MLE) of NPLD

The likelihood function of NPLD is given by

$$\begin{aligned} L(\mu ,\sigma ,k,\lambda )= \frac{k^{n}\lambda ^{kn}}{(2\pi \sigma ^{2})^{n/2}}e^{\sum _{i=1}^{n} \left\{ -\frac{1}{2 \sigma ^{2}}\left[ ln\left( \frac{x_{i}^{k}}{\lambda ^{k}-x_{i}^{k}}\right) -\mu \right] ^{2}\right\} } \\ \times \prod _{i=1}^{n}\frac{1}{x_{i}(\lambda ^{k}-x_{i}^{k})} \end{aligned}$$

Taking the log gives

68 $$\begin{aligned}&\ell = nlnk + knln \lambda - \frac{n}{2}ln(2\pi \sigma ^{2}) \nonumber \\&\quad -\frac{1}{2 \sigma ^{2}}\sum _{i=1}^{n} \left\{ \left[ ln\left( \frac{x_{i}^{k}}{\lambda ^{k}-x_{i}^{k}}\right) -\mu \right] ^{2}\right\} \nonumber \\&\quad -\sum _{i=1}^{n} ln x_{i}-\sum _{i=1}^{n} ln(\lambda ^{k}-x_{i}^{k}) \end{aligned}$$

The maximum likelihood estimation parameters of the NPLD are given by differentiating $\ell$ partially with respect to $\mu$, $\sigma$ and *k* and equating the results to zero and solve for each parameter.

69 $$\begin{aligned}&\frac{\partial \ell }{\partial \mu }= \frac{1}{ \sigma ^{2}}\sum _{i=1}^{n} \left[ ln\left( \frac{x_{i}^{k}}{\lambda ^{k}-x_{i}^{k}}\right) -\mu \right] \end{aligned}$$

70 $$\begin{aligned}&\frac{\partial \ell }{\partial \sigma }= - \frac{n}{\sigma } +\frac{1}{\sigma ^{3}}\sum _{i=1}^{n} \left\{ \left[ ln\left( \frac{x_{i}^{k}}{\lambda ^{k}-x_{i}^{k}}\right) -\mu \right] ^{2}\right\} \end{aligned}$$

71 $$\begin{aligned}&\frac{\partial \ell }{\partial k}= \frac{n}{k} - nln\lambda - \frac{\lambda ^{k}}{\sigma ^{2}} \sum _{i=1}^{n} \left[ ln \left( \frac{x_{i}^{k}}{\lambda ^{k}-x_{i}^{k}}\right) - \mu \right] \left( \frac{lnx_{i} -ln\lambda }{\lambda ^{k}-x_{i}^{k}}\right) \nonumber \\&\quad - \sum _{i=1}^{n}\left( \frac{\lambda ^{k}ln\lambda - x_{i}^{k} ln x_{i}}{\lambda ^{k}-x_{i}^{k}}\right) \end{aligned}$$

Equating (69) to zero gives

72 $$\begin{aligned} {\hat{\mu }}= \frac{1}{n\sigma ^{2}}\sum _{i=1}^{n} \left[ ln\left( \frac{x_{i}^{k}}{\lambda ^{k}-x_{i}^{k}}\right) \right] . \end{aligned}$$

Equating (70) to zero gives

73 $$\begin{aligned} {\hat{\sigma }} =\sqrt{\frac{1}{n}\sum _{i=1}^{n} \left\{ \left[ ln\left( \frac{x_{i}^{k}}{\lambda ^{k}-x_{i}^{k}}\right) -\mu \right] ^{2}\right\} } \end{aligned}$$

Equating (71) to zero gives

74 $$\begin{aligned}&\frac{n}{k}= nln\lambda - \frac{\lambda ^{k}}{\sigma ^{2}} \sum _{i=1}^{n} \left[ ln \left( \frac{x_{i}^{k}}{\lambda ^{k}-x_{i}^{k}}\right) - \mu \right] \left( \frac{lnx_{i} - ln\lambda }{\lambda ^{k}-x_{i}^{k}}\right) \nonumber \\&\quad + \sum _{i=1}^{n}\left( \frac{\lambda ^{k}ln\lambda - x_{i}^{k} ln x_{i}}{\lambda ^{k}-x_{i}^{k}}\right) \end{aligned}$$

The equations obtained by setting the partial derivatives $\ell$ with respect to *k* to zero is not in closed form and the values of the parameter *k* is found using Newton’s numerical procedure provided by R package (R Development Core Team 2009). The parameter $\lambda$ cannot be estimated using the MLE method because it depends on *X*, thus, is estimated from from data using

75 $$\begin{aligned} {\hat{\lambda }}= max(x_{i}) + \epsilon ; \qquad \forall \, x \in X \end{aligned}$$

where $\epsilon > 0$ is a very small positive number less than 1 chosen by the user.

It should be noted that the maximum likelihood estimators of the parameters $\mu$ and $\sigma$ are in close form and will always exist provided the values of parameters *k* and $\lambda$ are known. The value of parameter $\lambda$ cannot be determined by the maximum likelihood estimation method because it is an upper bound, so it can be estimated by equation (75) from the data. Parameter *k* is not in closed form and a numerical optimization method is used to estimate it. We find the initial value of *k* used in the numerical optimization by first assuming that the random sample is from power function distribution. We estimate the initial value of *k* from power function distribution. The moment estimate of parameter *k* is given by $k = \frac{{\bar{x}}}{\lambda - {\bar{x}}}, {\bar{x}}<\lambda$, where ${\bar{x}}$ is the sample mean (Ekum et al. 2020b), estimated from data.

### Numerical Optimization of Parameter *k*

In a case where the parameter estimated using Newton approximation is not optimal, a new relationship is derived by EM algorithm.

Let

76 $$\begin{aligned} k_{\iota + 1} = \arg \max _{k} \int f(x|I;\Omega _{\iota }) lnf(x,I;\Omega )dx \end{aligned}$$

where $\Omega$ is the parameter space of NPLD, so that we have

77 $$\begin{aligned} k_{\iota + 1} = \arg \max _{k} \int f(x|I;k) lnf(x,I;\mu , \sigma , k, \lambda )dx \end{aligned}$$

Recall the pdfs of normal distribution and NPLD as

$$\begin{aligned} f(x) = \frac{1}{\sqrt{2\pi \sigma ^{2}}}e^{-\frac{1}{2} \left( \frac{x-\mu }{\sigma } \right) ^{2}} \end{aligned}$$

and

$$\begin{aligned} f_{X}(x)= \frac{k \lambda ^{k}}{x(\lambda ^{k}-x^{k})\sqrt{2\pi \sigma ^{2}}} exp\left\{ -\frac{1}{2\sigma ^{2}} \left[ ln\left( \frac{x^{k}}{\lambda ^{k}-x^{k}} \right) -\mu \right] ^{2} \right\} \end{aligned}$$

respectively.

Substituting the pdfs of normal distribution and NPLD into Equation (77) gives

78 $$\begin{aligned}&k_{\iota + 1} = \arg \max _{k} \int _{0}^{\infty } \frac{1}{\sqrt{2\pi S^{2}}}e^{-\frac{1}{2} \left( \frac{x-{\bar{x}}}{S} \right) ^{2}} \nonumber \\&\times ln \left\{ \frac{k {\hat{x}}_{(n)}^{k}}{x({\hat{x}}_{(n)}^{k}-x^{k})\sqrt{2\pi S^{2}}} e^{\left\{ -\frac{1}{2S^{2}} \left[ ln\left( \frac{x^{k}}{{\hat{x}}_{(n)}^{k}-x^{k}} \right) -{\bar{x}} \right] ^{2} \right\} } \right\} dx. \end{aligned}$$

where $\mu$, $\sigma$ and $\lambda$ are known, such that, ${\hat{\mu }} = {\bar{x}}$, ${\hat{\sigma }}=S$, and ${\hat{\lambda }}= \sup _{x} = {\hat{x}}_{(n)}$, where ${\bar{x}}$ and *S* are the sample mean and sample standard deviation of $ln \left( \frac{x}{\lambda - x} \right)$. Note that ${\hat{x}}_{(n)} - x > 0, \, \forall \, x \in X$. Note $k_{1}$ is the initial value of *k* assumed as suggested, that is, $k_{1} = \frac{{\bar{x}}}{\lambda - {\bar{x}}}, {\bar{x}}<\lambda$. So that $k_{\iota + 1}$ is the new estimate of *k* and it is optimal.

Now that optimal value of *k* is known, then we can estimate the values of $\mu$ and $\sigma$ using equations (72) and (73) respectively.

### Error Bound and Confidence Interval for NPLD

The error bound for estimating a generic parameter $\Theta$ of NPLD is given by

79 $$\begin{aligned} B = Q^{*}_{(1-\alpha )}S_{\Theta } \end{aligned}$$

where $\alpha$ is the level of significance, $\Theta$ is the parameter to be estimated, $Q^{*}_{p}$ is the standard quantile function of NPLD with $p = 1 - \alpha ; p \in [0,1]$, and $S_{\Theta }$ is the standard error of $\Theta$, that is, the square root of the variance of $\Theta$.

The standard quantile function of NPLD is derived when $k = \sigma = 1$ and $\mu = 0$ from the quantile function of NPLD and it is given by

80 $$\begin{aligned} Q^{*}_{p} = \lambda \left( \frac{e^{\Phi ^{-1}(p)}}{1+\Phi ^{-1}(p)}\right) \end{aligned}$$

where $Q^{*}_{p}$ is the standard quantile function of NPLD, $\Phi ^{-1}(p)$ is the inverse function of the cdf of standard normal distribution known as the quantile function, and *p* is a probability value uniformly generated. Note that $\lambda > 0$ is a regulator parameter in this case. Its value is adjusted to determine how large the error bound should be. In this research, $\lambda$ is taken as 2 to accommodate the population parameter. So, the level of significance, $\alpha$ and $\lambda$ are always chosen. The values of $\lambda$ can be 1, 2 or 3 depending on how large you want the error bound to be.

Thus, the $100(1-\alpha ) \%$ confidence interval for parameter $\Theta$ is given by

81 $$\begin{aligned} \Theta = {\hat{\Theta }} \pm Q^{*}_{(1-\alpha )}S_{\Theta } \end{aligned}$$

where ${\hat{\Theta }}$ is the point estimate of $\Theta$.

### Simulation Study of NPLD

The simulation study is presented to show the performances of the maximum likelihood estimators and their consistency. The procedure used to perform the simulation studies involves, generating uniform distribution of *n* quantiles, *p*. The quantile function defined in equation (21) for NPLD was used to generate NPLD random variates for the sample sizes *n* = 50, 100, 200 and 300 replicated 1000 times. The parameters values are set as $k=\sigma =\mu = 0.5$ , $k=\sigma =\mu =1$, and $k=\sigma =\mu =2$ and for a fixed $\lambda = 2$. The actual values, mean estimates, standard errors, and 95% confidence interval are presented in Tables 1, 2 and 3. Tables 1, 2 and 3 show that the standard error decreases as the sample size increases, which implies that the MLEs are consistent.

**Table 1:** Simulation Study showing Mean estimates, standard error, and confidence interval of the MLE for $k = \sigma = \mu = 0.5$

*n*	Parameters	Actual values	Mean	Standard error	Confidence interval
50	*k*	0.5	0.5503	0.0503	(0.4660, 0.6346)
	$\sigma$	0.5	0.5005	0.1005	(0.3320, 0.6690)
	$\mu$	0.5	0.5488	0.1488	(0.2994, 0.7982)
100	*k*	0.5	0.5323	0.0203	(0.4983, 0.5663)
	$\sigma$	0.5	0.5004	0.1001	(0.3326, 0.6682)
	$\mu$	0.5	0.5286	0.1178	(0.3311, 0.7261)
200	*k*	0.5	0.5104	0.0102	(0.4933, 0.5275)
	$\sigma$	0.5	0.5002	0.0055	(0.491, 0.5094)
	$\mu$	0.5	0.5078	0.0468	(0.4293, 0.5863)
300	*k*	0.5	0.5003	0.0100	(0.4835, 0.5171)
	$\sigma$	0.5	0.4998	0.0014	(0.4975, 0.5021)
	$\mu$	0.5	0.5008	0.0088	(0.486, 0.5156)

**Table 2:** Simulation Study showing Mean estimates, standard error, and confidence interval of the MLE for $k = \sigma = \mu = 1$

*n*	Parameters	Actual values	Mean	Standard error	Confidence interval
50	*k*	1	1.0046	0.0158	(0.9781, 1.0311)
	$\sigma$	1	1.0019	0.0981	(0.8374, 1.1664)
	$\mu$	1	1.4142	0.3144	(0.8871, 1.9413)
100	*k*	1	1.0053	0.0141	(0.9817, 1.0289)
	$\sigma$	1	1.0014	0.0942	(0.8435, 1.1593)
	$\mu$	1	1.4018	0.2980	(0.9022, 1.9014)
200	*k*	1	1.0030	0.0032	(0.9976, 1.0084)
	$\sigma$	1	1.0012	0.0902	(0.8500, 1.1524)
	$\mu$	1	1.3154	0.2100	(0.9634, 1.6674)
300	*k*	1	1.0028	0.0028	(0.9981, 1.0075)
	$\sigma$	1	1.0011	0.0682	(0.8868 1.1154)
	$\mu$	1	1.4158	0.1158	(1.2217, 1.6099)

**Table 3:** Simulation Study showing Mean estimates, standard error, and confidence interval of the MLE for $k = \sigma = \mu = 2$

*n*	Parameters	Actual values	Mean	Standard error	Confidence interval
50	*k*	2	1.9986	0.1017	(1.8281, 2.1691)
	$\sigma$	2	2.0035	0.2965	(1.5065, 2.5005)
	$\mu$	2	2.1415	0.4451	(1.3953, 2.8877)
100	*k*	2	1.9987	0.0325	(1.9442, 2.0532)
	$\sigma$	2	2.0031	0.0964	(1.8415, 2.1647)
	$\mu$	2	2.0544	0.3547	(1.4598, 2.6490)
200	*k*	2	1.9992	0.0064	1.9885, 2.0099)
	$\sigma$	2	2.0026	0.0163	(1.9753, 2.0299)
	$\mu$	2	2.0066	0.0178	(1.9768, 2.0364)
300	*k*	2	2.0001	0.0060	(1.9900, 2.0102)
	$\sigma$	2	2.0011	0.0112	(1.9823, 2.0199)
	$\mu$	2	2.0018	0.0129	(1.9802, 2.0234)

### Generalized Linear Regression Model for NPLD (NPGLM)

Let assume that the dependent random variable *Y* of interest in our linear model follows a NPLD given independent variable(s) *X*. The linear regression model is called NPLD Generalized Linear Model (NPGLM).

Given the linear model in matrix form

82 $$\begin{aligned} Y = XB + e \end{aligned}$$

where *Y* is a *n*-dimensional vector called the dependent vector for all observations *n*; *X* is the set of *k* independent variables packed into a ($n \times k + 1$) matrix called the design matrix; *B* is a ($k+1$)-dimensional vector called the slope vector; *e* is the error term packed into a *n*-dimensional vector called the error vector.

#### Conditions for NPGLM

The conditions to use the GPGLM to fit the model are given thus:

- *Y* must be continuous random variable
- *Y* must be positive real number strictly greater than zero but strictly less than $\lambda$ (upper bound for *Y*)
- *Y* must follow NPLD
- NPLD must be a member of the exponential family

#### Exponential Class of NPLD

An exponential family or class is a parametric set of probability distributions that has a certain form. This special form is chosen for mathematical convenience, based on some useful algebraic properties, as well as for generality (Akarawak et al. 2017). It is assumed that each component of *Y* follows a distribution in the exponential family of the form

83 $$\begin{aligned} f_{Y}(y; \theta , \phi ) = c(T(y),\phi ) exp\left\{ \frac{\left[ \theta T(y) - b(\theta ) \right] }{a(\phi )}\right\} ; \, \, \, \, a(\phi )>0, \end{aligned}$$

where $a(\phi )$ is a function of a known parameter $\phi$ only, $b(\theta )$ is a function of a canonical parameter $\theta$ and $c(T(y),\phi )$ is a function of *y* and $\phi$ only, and *T*(*y*) is a function of *y*, known as the sufficient statistics for *Y*.

Let assume that *Y* is a random variable that follows NPLD. Recall the pdf of the NPLD with parameters $\mu , \sigma , k, \lambda$ given by

$$\begin{aligned} f_{X}(x)= \frac{k \lambda ^{k}}{x(\lambda ^{k}-x^{k})\sqrt{2\pi \sigma ^{2}}} exp\left\{ -\frac{1}{2\sigma ^{2}} \left[ ln\left( \frac{x^{k}}{\lambda ^{k}-x^{k}} \right) -\mu \right] ^{2} \right\} \end{aligned}$$

where parameter $\lambda$ is an upper bound. The pdf *f*(*y*) is not free from parameter ($\lambda$), and hence, might be difficult to express as a member of the exponential family.

However, a simple transformation can be done with the data that follows a NPLD to a normal distribution as proved in Lemma (2.1).

Recall the transformed pdf

84 $$\begin{aligned} f(w) =\frac{1}{\sqrt{2 \pi \sigma ^{2}}} exp \left\{ -\frac{1}{2} \left( \frac{w - \mu }{\sigma }\right) ^{2}\right\} \end{aligned}$$

Taking the log of (84) gives

85 $$\begin{aligned} log f(w) = -log (\sqrt{2 \pi })-log \sigma -\frac{w^{2}}{2\sigma ^{2}} -\frac{w\mu }{\sigma ^{2}} -\frac{\mu ^{2}}{2\sigma ^{2}} \end{aligned}$$

Taking the exponential of (85) gives

86 $$\begin{aligned} f(w) = exp\left[ -log (\sqrt{2 \pi })-log \sigma -\frac{w^{2}}{2\sigma ^{2}} \right] exp\left[ \frac{-w\mu - \mu ^{2}/2}{\sigma ^{2}} \right] \end{aligned}$$

Comparing (86) with (83) gives

$$\theta T(y) = -w\mu$$, $$T(y) = w$$, $$\theta = -\mu$$, $$b(\theta )= -\mu ^{2}/2$$, $$a(\phi ) = \phi$$, $$\phi = \sigma ^{2}$$ and $$c(T(y),\phi )=exp\left[ -log (\sqrt{2 \pi })-log \sigma -\frac{w^{2}}{2\sigma ^{2}} \right]$$,

where *w* is a function of $y, k, \lambda$ given by

$$\begin{aligned} w = - log \left( \frac{y^{k}}{\lambda ^{k}-y^{k}} \right) . \end{aligned}$$

Since (86) can be written in exponential class, we can directly derive the joint sufficient statistics from it. So, the joint sufficient statistics for $\mu$ and $\sigma$ are *w* and $w^{2}$ respectively. Thus, *w* and $w^{2}$ can give all information concerning parameters $\mu$ and $\sigma$ respectively.

#### Maximum Likelihood Estimation of the Parameters of NPLD Regression Model

The log-likelihood of the pdf of NPLD is

87 $$\begin{aligned} logL = -nlog (\sqrt{2 \pi })-nlog \sigma -\sum _{i=1}^{n}\frac{w_{i}^{2}}{2\sigma ^{2}} - \sum _{i=1}^{n}\frac{w_{i}\mu }{\sigma ^{2}} - n\frac{\mu ^{2}}{2\sigma ^{2}} \end{aligned}$$

The link function is given by

88 $$\begin{aligned} g(\mu _{i})= \mu _{i} = XB = b_{0}+b_{1}x_{1i}+...+b_{p}x_{pi} \end{aligned}$$

So that

89 $$\begin{aligned}&logL = -nlog (\sqrt{2 \pi })-nlog \sigma -\sum _{i=1}^{n}\frac{w_{i}^{2}}{2\sigma ^{2}} - \sum _{i=1}^{n}\frac{w_{i}x_{ij}^{'}b_{j}}{\sigma ^{2}} \nonumber \\&\quad - \sum _{i=1}^{n}\frac{(x_{ij}^{'}b_{j})^{2}}{2\sigma ^{2}} \end{aligned}$$

where $j = 0, 1,...,p$, $E(w_{i}) = \mu _{i} = x_{ij}^{'}b_{j}$ and $w_{i} = log\left( \frac{y_{i}^{k}}{\lambda ^{k}-y_{i}^{k}} \right)$. Note that *p* is the number of independent variables, and $\alpha$, *k* and $\lambda$ are known parameters.

Then

90 $$\begin{aligned}&logL = -nlog (\sqrt{2 \pi })-nlog \sigma -\sum _{i=1}^{n}\frac{\left[ log\left( \frac{y_{i}^{k}}{\lambda ^{k}-y_{i}^{k}} \right) \right] ^{2}}{2\sigma ^{2}} \nonumber \\&\quad - \sum _{i=1}^{n}\frac{\left[ log\left( \frac{y_{i}^{k}}{\lambda ^{k}-y_{i}^{k}} \right) \right] x_{ij}^{'}b_{j}}{\sigma ^{2}} - \sum _{i=1}^{n}\frac{(x_{ij}^{'}b_{j})^{2}}{2\sigma ^{2}} \end{aligned}$$

The MLE parameter estimate for $b_{j}$ is in closed form and it is given by

91 $$\begin{aligned} {\hat{B}} = (X'X)^{-1}X'\left[ log\left( \frac{y_{i}^{k}}{\lambda ^{k}-y_{i}^{k}} \right) \right] ; \lambda> y \, \forall \, y \in Y, \, k>0. \end{aligned}$$

where ${\hat{B}} = [{\hat{b}}_{0}, {\hat{b}}_{1},...,{\hat{b}}_{p}]$, $X = [X_{1}, X_{2},...,X_{p}]$ is a $(n \times p)$ matrix, so that $X'$ is a $(p \times n)$ matrix, *W* is an $(n \times 1)$ matrix, so that $X'W$ is a $(p \times 1)$ matrix. Thus, ${\hat{B}}$ is a ($p\times 1$) matrix. Note that $W = log\left( \frac{Y^{k}}{\lambda ^{k}-Y^{k}} \right)$, where the value of lambda can be approximated from the data using nth order statistic or simply ${\hat{\lambda }} = max(y_{i})+\sigma _{{\bar{y}}}$ $\forall$ *i*, where $\sigma _{{\bar{y}}}$ is the standard error of *y* computed from the data. An approximation for *k* can also be derived from data using ${\hat{k}}= \frac{{\bar{y}}}{\lambda - {\bar{y}}}, {\bar{y}}<\lambda$, where ${\bar{y}}$ is the sample mean, derived from Ekum et al. (2020b).

## Results

### Application

In this section, applications to three real data sets were provided to illustrate the uses and importance of the NPLD. Three competing models are used to fit the two data of interest, they are NPLD, Normal are Gamma GLMs.

#### Application 1: Estimated Spill Volume (ESV) of Crude Oil in Nigeria

The data on the estimated spilled volume (ESV) is collected from 7th January 2011 to 27th December 2019, at Shell Nigeria webisite (www.shell.com.ng/sustainability/environment/oil-spills.html).

**Fig. 2:**
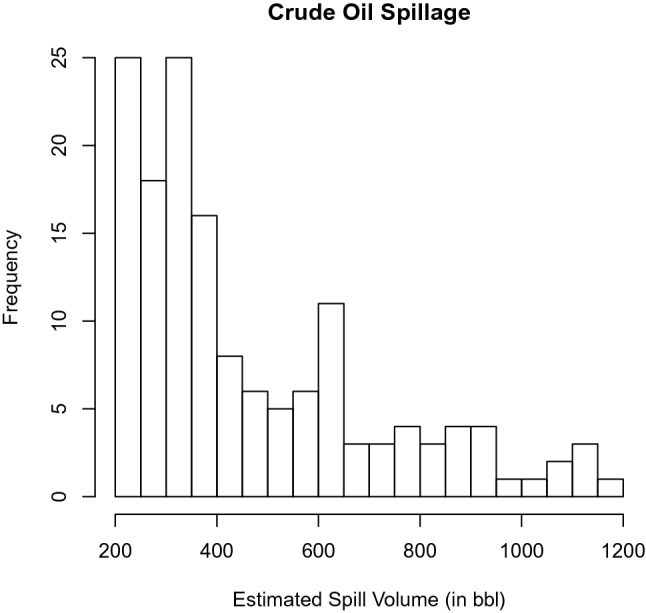
Histogram showing ESV of Crude Oil in Nigeria

Figure 2 shows that the oil spill data is bimodal with positive skewness (1.1302) and kurtosis (3.3977).

#### Fitting the Models to Oil Spill Data

The estimated spill volume of crude oil can be determined by the Duration of Clean-up (DOC). If the duration of clean-up is known, the spill volume can be estimated from an appropriate model. Thus, the dependent variable is ESV and the independent variable is the DOC.

**Table 4:** Generalised linear model parameter estimates for Oil Spill model

Distribution	${\hat{\beta }}_{0}$	$SE_{{\hat{\beta }}_{0}}$	*P*-Value	${\hat{\beta }}_{1}$	$SE_{{\hat{\beta }}_{1}}$	*P*-Value
NPLD	1.463495	0.000920	0.000000	0.007670	0.000002	0.000000
Normal	-87.909300	60.111900	0.147000	4.101300	0.807100	0.000002
Gamma	0.019900	0.004512	0.000029	-0.000071	0.000016	0.000042

Table 4 shows the model parameters estimated, their standard errors and their corresponding *P*-values.

**Table 5:** Generalised linear model goodness-of-fit criteria for ESV model

Distribution	$-LogL$	*AIC*	*D*	*A*	$\omega$	$\chi ^{2}$
NPLD	93.97682	193.9536	0.2809	6.0198	3.4428	43.060
Normal	663.5405	1333.0810	0.4157	6.0410	4.0625	1558.443
Gamma	401.7506	809.5012	0.6966	10.6512	8.1603	370.830

Table 5 shows that the NPLD regression model outperforms the other regression models using all the selection criteria.

#### Application 2: Total Research Gate Score

**Fig. 3:**
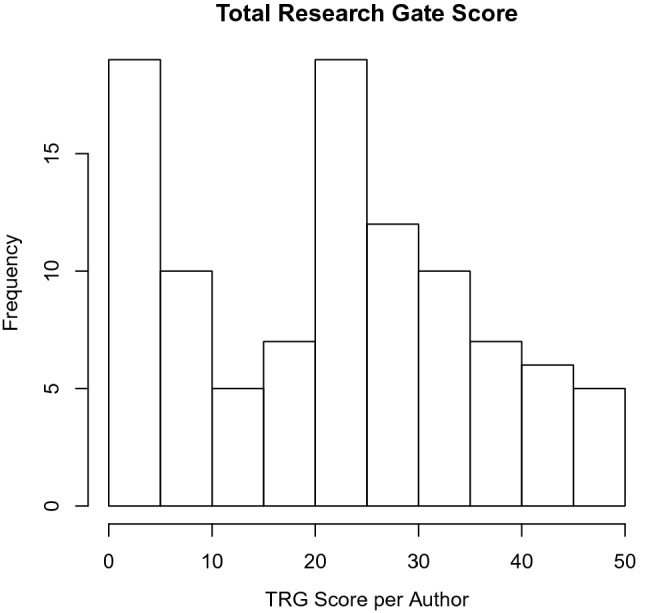
Histogram showing TRG Score of some selected researchers

Total Research Gate (TRG) score data is a cross-sectional data collected from Research Gate page of 100 selected researchers in the field of Mathematical Science as of 15th May 2021. The data includes TRG score, Total Research Interest (TRI), Citations, Recommendations, Reads and Research Items (RI). The independent variables are citations and RI (Fig. 3).

Figure 3 shows that the TRG score data is bimodal with positive skewness of 0.1595 and kurtosis of 1.9747.

#### Fitting the Models to Research Gate Data

The TRG score can be predicted by Citations and Research Items. If citations and research items increased, the TRG score will also increase. Thus, the dependent variable is TRG score, while the independent variables are citations and research items.

**Table 6:** Generalised linear model parameter estimates for TRG Score model

Distribution	${\hat{\beta }}_{1}$	$SE_{{\hat{\beta }}_{1}}$	*P*-Value	${\hat{\beta }}_{2}$	$SE_{{\hat{\beta }}_{2}}$	*P*-Value
NPLD	0.00212	0.00000	0.00015	0.05554	0.00000	0.00000
Normal	0.00117	0.00036	0.00151	0.04910	0.00857	0.00000
Gamma	-0.00002	0.00000	0.00022	-0.00003	0.00000	0.00030

Table 6 shows the model parameters estimated using MLE, their standard error and their corresponding *P*-values. The fitted NPLD regression model shows that the estimates $\beta _{0}$ and $\beta _{1}$ are significant at 5$\%$ level of error. This is also true for gamma and normal regression models.

**Table 7:** Generalised linear model goodness-of-fit criteria for TRG Score model

Distribution	$-LogL$	*AIC*	*D*	*A*	$\omega$	$\chi ^{2}$
NPLD	280.5727	565.1455	0.2145	9.0244	1.5532	10.5850
Normal	379.0210	762.0421	0.2305	10.5501	1.9402	94.1980
Gamma	344.9956	693.9912	0.3247	17.9003	3.6227	58.6690

Table 7 shows that the NPLD regression model outperforms the other regression models using all the goodness-of-fit criteria.

#### Application 3: Gross Domestics Product per Capita per COVID-19 Cases

The data used here are daily data collected from World Health Organisation (WHO) from 1st June 2020 to 31st December 2020, spanning 214 datasets, used by Iluno et al. (2021). The independent variable is a measure of COVID-19, termed COVID-19 Mortality per 1 million persons in the population (CMP), while the dependent variable is the GDP per capita per COVID-19 laboratory-confirmed cases (RGDPC). The CMP is a proxy to measure COVID-19 mortality, while RGDPC is a proxy to measure the economic wellbeing of a country.

**Fig. 4:**
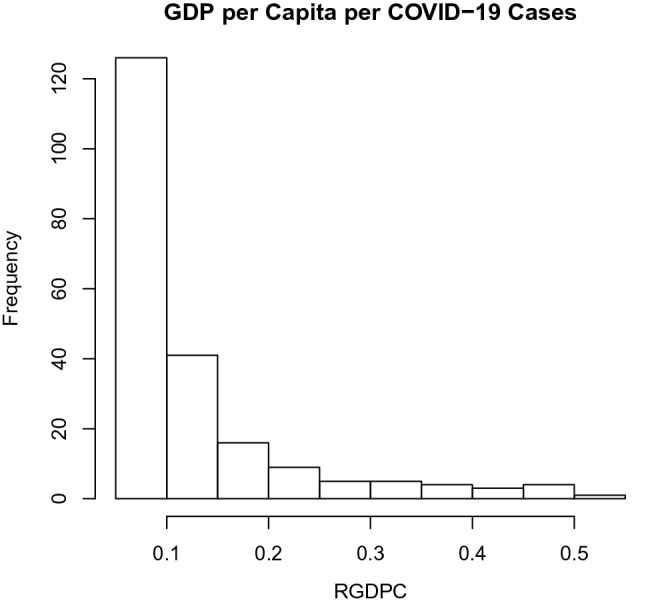
Histogram showing RGDPC of Nigeria

Figure 4 shows that the RGDPC data has a positive skewness of 2.317554 and kurtosis of 7.896267. This data is highly skewed and very peaked (leptokurtic).

#### Fitting the Models to COVID-19 Data

The RGDPC can be predicted by the CMP. If COVID-19 Mortality per Population is high, it can affect the GDP per Capita of a country negatively. Thus, the dependent variable is RGDPC and the independent variable is the CMP. Four competing distributions are used to fit the GLM. The performance of the three competing models are presented in Table 8 to show the performance of the models when fitted to the RGDPC data (Table 9).

**Table 8:** Generalised linear model parameter estimates for RGDPC model

Distribution	${\hat{\beta }}_{0}$	$SE_{{\hat{\beta }}_{0}}$	*P*-Value	${\hat{\beta }}_{1}$	$SE_{{\hat{\beta }}_{1}}$	*P*-Value
NPLD	3.01647	0.00080	0.00000	−0.53761	0.00058	0.00000
Normal	0.45155	0.00890	0.00000	−0.06810	0.00184	0.00000
Gamma	−1.92291	0.06327	0.00000	2.44210	0.02002	0.00000

Table 8 shows the model parameters estimated, their standard errors and their corresponding *P*-values.

**Table 9:** Generalised linear model goodness-of-fit criteria for RGDPC model

Distribution	$-LogL$	$AIC$	*D*	$A$	$\omega$	*χ*2
NPLD	280.5727	565.1455	0.2145	9.0244	1.5532	10.5850
Normal	379.0210	762.0421	0.2305	10.5501	1.9402	94.1980
Gamma	344.9956	693.9912	0.3247	17.9003	3.6227	58.6690

Table 9 shows that the NPLD regression modeloutperforms the other regression models using all the selection criteria.

## Conclusions

This study developed a novel NPLD model, using the T-Power$\lbrace$logistic$\rbrace$ family of distributions. The cdf, pdf, survival function, hazard rate, cumulative hazard function, reverse hazard function, useful transformation, quantile functions, mode, robust skewness, robust kurtosis, series expansion and moment are derived. The maximum likelihood estimation of the parameters of the distribution were derived and that of its generalized regression model. The NPLD regression model was applied to three real-life data namely, Estimated Spill Volume (ESV) of crude oil in Niger Delta area of Nigeria, Total Research Gate (TRG) score of some selected researchers in research gate and GDP per Capita per COVID-19 cases (RGDPC; and the results of its performance was compared favourably with normal and Gamma regression models.

The goodness of fit statistics showed that the NPLD regression model outperforms the other regression models using all the selection criteria. Also, the goodness of fit statistics also show that the NPLD regression model outperforms the other regression models using all the criteria for the TRG score model as well as the RGDPC model. Hence, NPLD regression model can be used effectively to analyze and model the crude oil spill volume data, TRG score data, RGDPC and other related data when normal is not good fit.

This research therefore recommends that

- NPLD model should be used to estimate spill volume of crude oil, and total research gate score.
- It is recommended that the convoluted distribution NPLD should be used when normal is not a good fit to emerging data of interest.
- It is recommended based on the applications that clean-up of spilled oil should be carried out immediately and complete it at record time, because it can be used to estimate the spilled volume of crude oil.
- It is also recommended that researchers should increase the research items they upload to research gate and write quality papers to increase their citations, in order to increase their total research gate score.
- It is also recommended that COVID-19 mortality be reduced, by providing medical response to infected individuals, because, it can affect the economic well-being of the nation.
